# Improved Survival in Patients with Viral Hepatitis-Induced Hepatocellular Carcinoma Undergoing Recommended Abdominal Ultrasound Surveillance in Ontario: A Population-Based Retrospective Cohort Study

**DOI:** 10.1371/journal.pone.0138907

**Published:** 2015-09-23

**Authors:** Hla-Hla Thein, Michael A. Campitelli, Latifa T. Yeung, Ahmad Zaheen, Eric M. Yoshida, Craig C. Earle

**Affiliations:** 1 Dalla Lana School of Public Health, University of Toronto, Toronto, Ontario, Canada; 2 Ontario Institute for Cancer Research/Cancer Care Ontario, Toronto, Ontario, Canada; 3 Institute for Clinical Evaluative Sciences, Toronto, Ontario, Canada; 4 Rouge Valley Health System, Scarborough, Ontario, Canada; 5 Department of Paediatrics, University of Toronto, Toronto, Ontario, Canada; 6 Institute of Health Policy, Management and Evaluation, University of Toronto, Toronto, Ontario, Canada; 7 Faculty of Medicine, University of Toronto, Toronto, Ontario, Canada; 8 University of British Columbia, Division of Gastroenterology, Vancouver, British Columbia, Canada; National Yang-Ming University, TAIWAN

## Abstract

The optimal schedule for ultrasonographic surveillance of patients with viral hepatitis for the detection of hepatocellular carcinoma (HCC) remains unclear owing to a lack of reliable studies. We examined the timing of ultrasonography in patients with viral hepatitis-induced HCC and its impact on survival and mortality risk while determining predictors of receiving surveillance before HCC diagnosis. A population-based retrospective cohort analysis of patients with viral hepatitis-induced HCC in Ontario between 2000 and 2010 was performed using data from the Ontario Cancer Registry linked health administrative data. HCC surveillance for 2 years preceding diagnosis was assigned as: i) ≥2 abdominal ultrasound screens annually; ii) 1 screen annually; iii) inconsistent screening; and iv) no screening. Survival rates were estimated using the Kaplan-Meier method and parametric models to correct for lead-time bias. Associations between HCC surveillance and the risk of mortality after diagnosis were examined using proportional-hazards regression adjusting for confounding factors. Overall, 1,483 patients with viral hepatitis-induced HCC were identified during the study period; 20.2% received ≥1 ultrasound screen annually (routine surveillance) for the 2 years preceding diagnosis. The 5-year survival of those receiving routine surveillance was 31.93% (95% CI: 25.77–38.24%) and 31.84% (95% CI: 25.69–38.14%) when corrected for lead-time bias (HCC sojourn time 70 days and 140 days, respectively). This is contrasted with 20.67% (95% CI: 16.86–24.74%) 5-year survival in those who did not undergo screening. In the fully adjusted model, compared to unscreened patients, routine surveillance was associated with a lower mortality risk and a hazard ratio of 0.76 (95% CI: 0.64–0.91) and 0.81 (95% CI: 0.68–0.97), corrected for the respective lead-time bias. Our findings suggest that routine ultrasonography in patients with viral hepatitis is associated with improved survival and reduced mortality risk in a population-based setting. The data emphasizes the importance of surveillance for timely intervention in HCC-diagnosed patients.

## Introduction

Hepatocellular carcinoma (HCC) is an increasing global public health problem, representing the sixth most common cancer and third most frequent cause of cancer-related death worldwide [1]. It is amongst the fastest growing diagnosed cancers in Canada [2–4], with incidence rates increasing in both males (3.4% per year) and females (2.2% per year) over the past 30 years with similar trends in mortality rates [2,3]. Over 80% of HCC worldwide is attributable to liver injury caused by chronic hepatitis B virus (HBV) or hepatitis C virus (HCV) infection [5–7], with the vast majority of HCC developing in the presence of underlying cirrhosis (80–90%) [8–14], In Europe and North America, HCV-related cirrhosis is the major underlying cause of HCC [7] with an annual incidence of 3–5% [15,16] and is the leading cause of death (~50%) [15,17]. The incidence of HCC is expected to continue to increase due largely to failed containment of hepatitis C, the aging Canadian population, and the increasing prevalence of obesity and diabetes mellitus amongst other persistent risk factors including excessive alcohol consumption and smoking [2,3].

Historically, most patients with HCC are diagnosed at an advanced stage of disease, often presenting with constitutional symptoms, liver function impairment, and/or extrahepatic metastasis. The prognosis after HCC diagnosis is poor, with a 5-year survival estimate of approximately 7% [18]. Diagnosis of the disease at an early stage, however, provides a role for potentially curative interventions including surgical resection, liver transplantation, and locoregional therapies such as radiofrequency ablation. These interventions have a significant impact on patient outcome with an improvement in 5-year survival by more than 50% [7,17,19–28]. In a cohort study of Child’s class A and B cirrhotic patients, semi-annual surveillance increased the detection rate of early stage HCC and reduced the number of advanced tumors when compared to an annual surveillance regimen. Furthermore, the cohort that underwent more rigorous surveillance benefited from increased survival when adjusted for lead-time bias [28]. The utility of regular surveillance is not without controversy as demonstrated by a recent systematic review that suggested the evidence for regular surveillance is associated with a survival benefit that is “very low level” given methodologic weaknesses of published studies and lead- and length-time biases [29].

Currently, practice guidelines from the American Association for the Study of Liver Diseases (AASLD), the European Association for the Study of the Liver (EASL), and the multidisciplinary Canadian consensus recommendations for the management and treatment of HCC [30] suggest surveillance for patients at high-risk for HCC to detect cancer at an early stage when it is amenable to potentially curative therapy [10]. Despite the obvious benefits of early intervention in HCC and the lack of alternative treatments in advanced disease, surveillance measures are not routinely implemented [31–33]. A recent Canadian study found that patients with HCC referred to a tertiary liver treatment center were more likely to be in palliative stages than those whose tumor was detected internally [34]. These results imply ineffective surveillance practice in the community setting, which may be responsible for disparate health outcomes [34]. Indeed, there are limited data on the utilization and patterns of recommended HCC surveillance in Canada. The objectives of this study were to: i) examine the timing of ultrasonographic surveillance and their impact on survival and mortality risk; and ii) determine predictors of receiving ultrasonographic surveillance before an HCC diagnosis in patients with viral hepatitis-induced HCC in Ontario. The data was obtained from the Ontario Cancer Registry (OCR) linked health administrative data which has the highest number of documented HCC cases in Canada [35].

## Materials and Methods

### Study design, setting, and population

**Fig 1:**
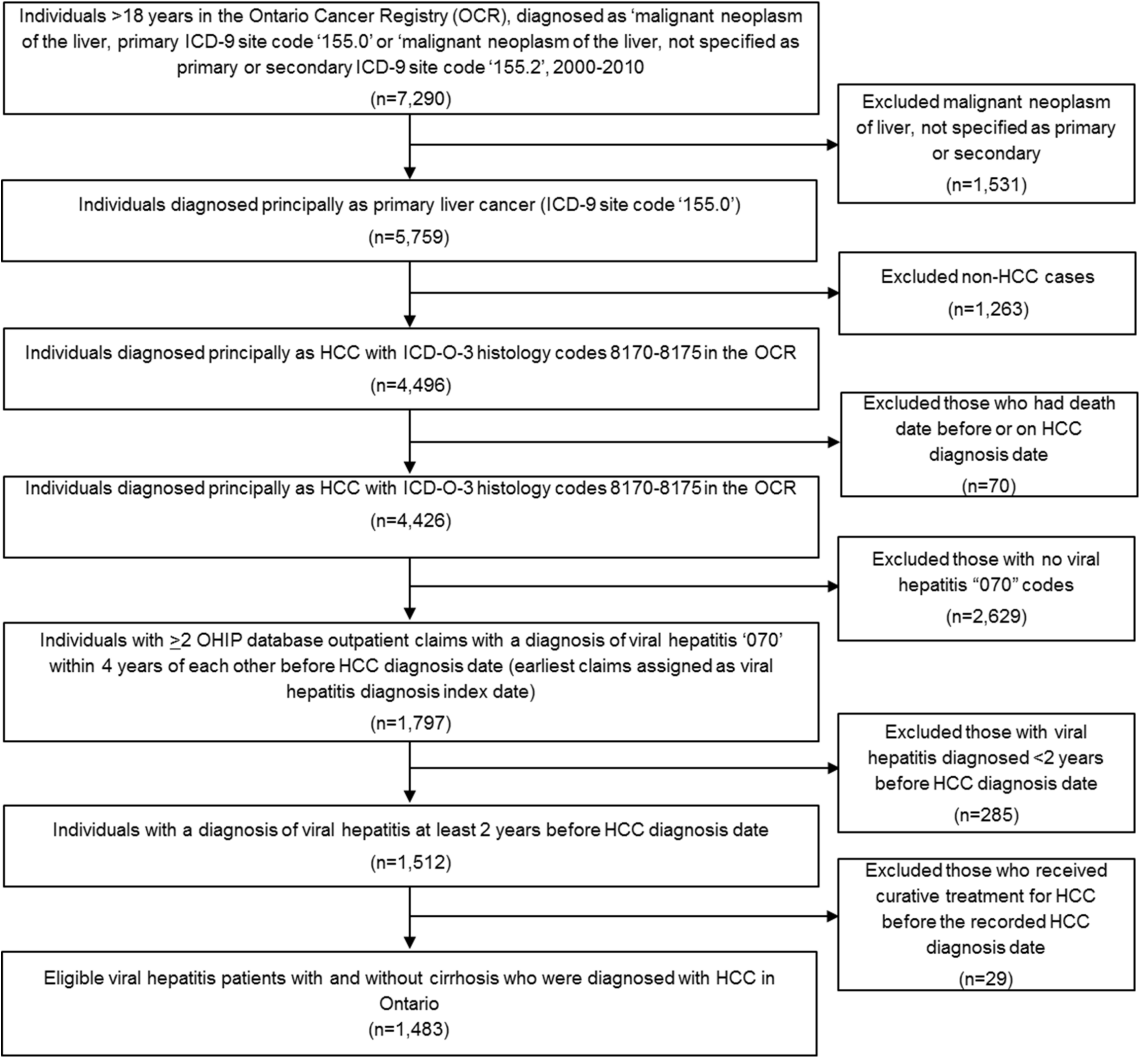
Study flow diagram.

We conducted a population-based retrospective cohort study of all eligible viral hepatitis patients aged 18 years and older with and without cirrhosis who were diagnosed with HCC in Ontario between January 1, 2000 and December 31, 2010. The International Classification of Diseases, 9th Revision (ICD-9) site code 155.0 was used to identify primary hepatic neoplasms in addition to the International Classification of Diseases for Oncology, Third Edition (ICD-O-3) histology codes 8170–8175. Patients were followed from the viral hepatitis index date to the date of their death or until the end of the study period (December 31, 2010). Patients who had death dates before or on the HCC diagnosis date were excluded. Of the remaining patients, those with at least two Ontario Health Insurance Plan (OHIP) diagnostic codes for viral hepatitis “070” within 4 years of each other before the HCC diagnosis date (the earliest claims assigned as viral hepatitis index date) and diagnosed at least 2 years before the HCC diagnosis were identified. Cases were identified only using ICD-9 coding due to the lack of ICD-10-CM B15-B19 code in the dataset. Those diagnosed with viral hepatitis less than 2 years before HCC diagnosis were excluded. Those who received potentially curative HCC treatment before the recorded HCC diagnosis date were also excluded. The selection criteria for the study population can be found in Fig 1. Based on a previous retrospective cohort study by Yeung [36] that aimed to validate health administrative data for the detection of HCV infection between 1 January 1995 and 31 March 2000, an algorithm consisting of more than one OHIP diagnostic code over a 5-year period had a sensitivity of 62.9%, specificity of 82.4%, positive predictive value of 54.9%, and negative predictive value of 86.8%. This suggests that more than half of all persons identified in the OHIP database utilizing this diagnostic code of viral hepatitis “070” will have a diagnosis of hepatitis C. Since hepatitis C and B represent the most common conditions of viral hepatitis [37], it is likely that hepatitis B accounts for the majority of the remaining cohort. For this study, the 4-year interval of viral hepatitis diagnosis before HCC diagnosis was chosen to be more conservative than the total 5-year interval studied.

### Data sources

Details of data sources are published elsewhere [35,38,39]. The OCR is a population-based cancer registry that collects data on incident cases of all tumors (except non-melanoma skin cancers) in Ontario since 1964 [40–43]. The OCR was linked to the OHIP database, the Discharge Abstract Database of the Canadian Institute for Health Information (CIHI), the Ontario Drug Benefit (ODB) program database, and the Canadian census data to provide individual-level information on sociodemographic, screening, treatment, and clinical factors [44]. The OHIP is a publically funded healthcare program for all Ontario residents. OHIP physician billing claim datasets contain service and diagnosis information for outpatient visits in Ontario. The CIHI Discharge Abstract Database contains information pertaining to diagnosis and procedures for all acute and chronic care hospitalizations in Ontario. The ODB dataset contains information regarding prescription medications (including sorafenib) dispensed to all adults aged ≥65 years and those receiving social assistance. The 1991, 1996, 2001, and 2006 Canadian census data were used to gather information on the socioeconomic variable of neighborhood income quintile (1, lowest and 5, highest) [35,45].

### HCC surveillance

We identified all abdominal ultrasonography performed on patients with viral hepatitis before HCC diagnosis utilizing OHIP fee codes such as diagnostic ultrasound-abdomen-abdominal scan-limited study (J128); diagnostic ultrasound-abdomen/retroperitoneal abdominal scan complete (J135); diagnostic ultrasound-abdomen & retroperitoneal.p2-abdominal scan-limited study (J428); diagnostic ultrasound-abdomen & retroperitoneal.p2-abdominal scan complete (J435). The timing of HCC ultrasonographic surveillance (at least 4.5 months apart from previous ultrasound) were assigned hierarchically as follows: i) ≥2 abdominal ultrasounds within 12 months and between 12-<24 months before HCC diagnosis (i.e., ≥2 screens annually for 2 years before HCC diagnosis); ii) 1 screen annually for 2 years before HCC diagnosis; iii) at least 1 screen either within 12 months or between 12-<24 months before HCC diagnosis (i.e., inconsistent screening); and iv) no screening before HCC diagnosis.

### Outcome measures

The main outcome for our study was survival time after diagnosis of HCC to death with correction for lead-time bias. The secondary outcomes were: i) the association between different ultrasonographic surveillance strategies and mortality risk after HCC diagnosis; and ii) predictors of receiving ultrasonographic surveillance before HCC diagnosis.

### Study variables

The OCR includes information on age at diagnosis, sex, cause of death, date of death, diagnosis date, postal code, and rural residence (classified by whether or not people were living in communities with less than 10,000 inhabitants) [46]. The Charlson-Deyo Comorbidity Index (comorbidity index value) was calculated using the methods previously described [47,48]; an ICD-9 coding algorithm was applied to the diagnostic field codes from the hospitalization data (excluding diagnoses of liver cirrhosis, alcoholic liver disease, metastatic cancer, diabetes). Baseline comorbidity was determined using the hospitalization records from the date of diagnosis. Conditions were weighted and then summed up to provide an overall comorbidity index value for a given episode, which was then categorized into one of five groups (0, 1, 2, ≥3, or no hospitalization record) representing different degrees of comorbidity. If cases did not have a hospitalization record at diagnosis date, we determined baseline comorbidity by looking back 2 years into the hospitalization data to find the most recent hospitalization record and applying the comorbidity score from that hospitalization [35]. Patients were assigned as having a missing comorbidity index value at baseline if they had no hospitalization records at diagnosis or 2 years before diagnosis. Comorbidity was adjusted for each hospitalization after baseline. Patients diagnosed with diabetes mellitus were identified from the CIHI and OHIP databases by the presence of ICD-9 code “250” and ICD-10 codes “E10-E14.” Covariates that denote liver disease severity measured before HCC diagnosis were also identified from the CIHI and OHIP databases: cirrhosis (ICD-9 code “571”; ICD-10 “K74”; OHIP “571”); alcoholic liver disease (ALD, ICD-9 “571.0”, “571.1”, “571.2”, “571.3”; ICD-10 “K70”); non-alcoholic fatty liver disease (NAFLD) (ICD-9 “571.8”; ICD-10 “K76.0); ascites (ICD-9 “789.5”; ICD-10 “R18”); esophageal varices (ICD-9 “456.0”, “456.1”, “456.2”; ICD-10 “I85”); and hepatic encephalopathy (ICD-9 “572.2”; ICD-10 “K72”). Subsequently, indicators of severe liver disease were categorized exclusively as: 1) no ALD+no cirrhosis; 2) no ALD+cirrhosis; 3) no ALD+decompensated cirrhosis (i.e., cirrhosis and any recorded ascites, esophageal varices, or hepatic encephalopathy); 4) ALD+no cirrhosis; 5) ALD+cirrhosis; 6) ALD+decompensated cirrhosis; and 7) NAFLD. Potentially curative treatment for HCC was considered as liver resection, liver transplantation, or radiofrequency ablation. Non-curative treatment was considered as chemotherapy or transarterial chemoembolization. Palliative treatment was defined as supportive management only. Codes used to identify HCC treatment can be found elsewhere [35].

### Statistical analysis

For each ultrasonographic surveillance scenario, sociodemographic and clinical characteristics, index year of HCC diagnosis, and HCC treatment were summarized as frequencies (percentages). HCC survival was calculated from the date of HCC diagnosis to the earliest of either the date of death or the end of the study period, 31 December 2010. Median survival times (days, with 95% confidence intervals [CI]), 1-year, 3-year, and 5-year survival after HCC diagnosis for patients receiving routine surveillance, inconsistent screening, and unscreened patients were estimated using the Kaplan-Meier method and compared using the log-rank test. A stepwise Cox proportional-hazards regression analysis was used to assess the effect of timing of HCC ultrasonographic surveillance on the risk of mortality and the explanatory effect of HCC curative treatment on potential effects of HCC surveillance; the first model measured unadjusted hazard ratios (HR) for the screening; the second model measured adjusted HR (aHR), adjusting for age and sex; the third model measured aHR, adjusting for sociodemographic characteristics (age, sex, rural residence, income quintile), clinical characteristics (comorbidity index value, diabetes mellitus diagnosis, indicators of severe liver disease, outpatient visits in the 2 years before HCC diagnosis), and index year of HCC diagnosis; and the final model measured aHR, adjusting for all covariates, including receipt of HCC curative treatment. Variables modeled as time-dependent covariates include comorbidity index value, diabetes diagnosis, and HCC curative treatment. The proportional-hazards assumption was assessed via a residual based test as described by Grambsch and Therneau [49]. The overall model fit was examined by plotting the Nelson-Aalen cumulative hazards estimates against Cox-Snell residuals and assessing their approximate adherence to the line of equality.

To correct lead-time bias for HCC ultrasonographic surveillance scenarios compared to non-screening, we used Schwartz’s formula [50], originally proposed for calculating tumor growth: *t* = D_T_ × 3 × log(d_U_/d_S_)/log(2), where *t* is the lead-time (days), D_T_ is the median value of the tumor volume doubling time proposed by Sheu *et al*. [51], and d_U_ and d_S_ are median tumor diameters of unscreened and screened patients, respectively. We applied D_T_ = 117 days (median, range 29–398 days) [51], d_U_ = 4.0 cm (median, range 0.5–16.0) [24], and d_S_ = 2.8 cm (median, range 0.7–16.0) [24,52] to calculate lead-times. The calculated lead-times ranged from 42 days (1.4 months) to 614 days (20.2 months). For the correction of lead-time bias for the above outcome measures, the survival time of patients who received surveillance was analyzed by applying a parametric model proposed by Duffy *et al*. [53] assuming an exponential distribution of the sojourn time, the preclinical screen-detectable period, with a rate of transition to symptomatic disease λ. Thus, 1/λ is the mean sojourn time. Correction for lead-time bias involves estimation of the additional follow-up time observed purely as a result of lead-time in a case of screen-detected cancer. The expected additional follow-up time, *s*, due to lead-time, that is: $E(s)=\frac{1-e^{-\lambda t}-{\lambda te}^{-\lambda t}}{\lambda (1-e^{-\lambda t})}$ for a patient with surveillance known to be dead at time *t*; and $E(s)=\frac{1-e^{-\lambda t}}{\lambda }$ for a patient with surveillance known to be alive at time *t* [53]. The lead-time was corrected by subtracting *E(s)* from the observed survival time of screen-detected cases. We assumed an average HCC sojourn time (1/λ) of 70 or 140 days (2.3 or 4.6 months), based on previous published reports [28,54]. A sensitivity analysis using a range of lead-time bias from 42 days to 614 days was performed to estimate outcomes.

Log binomial regression models were constructed to determine predictors (age, sex, rural residence, income quintile, baseline comorbidity index value, diabetes diagnosis, indicators of severe liver disease, outpatient visits, and viral hepatitis index year) [33] of receiving ≥1 ultrasound screening annually for 2 years before HCC diagnosis. Variables of known clinical importance with a univariate likelihood ratio test for the significance of the risk ratio (RR) at 0.20 level were initially chosen for inclusion in the multivariable log binomial regression model. The adjusted model was constructed according to a stepwise backward elimination, subject to a likelihood ratio test and only included those variables that remained significant at the 0.05 level (*p ≤ 0*.*05)*. Finally, variables that were non-significant in the univariate test were added to see if they became significant when adjusted for other factors.

### Ethics approval

Ethics approval for the study was granted by the University of Toronto Health Sciences Research Ethics Board. Informed consent was not obtained because this was secondary analysis of existing, de-identified data and ‎so consent was deemed not to be feasible or necessary.

## Results

Of individuals >18 years diagnosed liver cancer (n = 7,290), we identified 1,797 individuals with two or more OHIP database claims including a diagnosis of viral hepatitis within 4 years of each other before an HCC diagnosis from 2000 to 2010 (Fig 1). The final study cohort comprised 1,483 patients with a diagnosis of viral hepatitis at least 2 years before the HCC diagnosis date after excluding 285 patients with viral hepatitis diagnosed less than two years before HCC diagnosis date and a further 29 patients who received curative treatment for HCC before the recorded HCC diagnosis date. The mean, median and range of follow-up time of patients diagnosed with viral hepatitis were 3,920 days, 3,862 days, and 763–7,777 days, respectively. Of the total study cohort, 215 (14.5%) had a diagnosis of no ALD+cirrhosis only, 47 (3.2%) had ALD+cirrhosis, 350 (23.6%) had no ALD+decompensated cirrhosis, and 134 (9.0%) had ALD+decompensated cirrhosis (Table 1). The majority (n = 718, 48.4%) of HCC diagnosed patients were aged <60 years, male (n = 1,164, 78.5%), and urban residents (94.6%). Among the cohort with a viral hepatitis diagnosis, 20.4% (n = 302) received 1 screen or ≥2 abdominal ultrasound screens annually (routine surveillance) for 2 years before HCC diagnosis; of whom, the majority were patients diagnosed with no ALD+no cirrhosis (n = 156, 51.7%), no ALD+decompensated cirrhosis (n = 73, 24.2%), and no ALD+cirrhosis (n = 49, 16.2%; Table 1). The proportion of patients receiving potentially curative treatment was significantly higher in those who received screening compared with no screening (59.3% vs. 43.1%, *p*<0.001). The proportion of patients receiving potentially curative treatment was also higher among those receiving routine surveillance than those who received inconsistent screening (59.3% vs. 45.6%, *p*<0.001), but there was no significant difference between those receiving 1 screen and ≥2 screens (59.0% vs. 64.7%, *p* = 0.639).

**Table 1:** Descriptive characteristics of viral hepatitis patients diagnosed with hepatocellular carcinoma by different timing of ultrasonographic surveillance.

Characteristic	Total	No screening	Inconsistent screening*	1 screen^†^	≥2 screens^‡^	*P*-value
		N (%)	N (%)	N (%)	N (%)	
Overall	1,483	540 (36.41)	641 (43.22)	285 (19.22)	17 (1.15)	
Age at HCC diagnosis (years)						
*<60*	718 (48.42)	276 (51.11)	303 (47.27)	129 (45.26)	10 (58.82)	
*60–69*	414 (27.92)	163 (30.19)	171 (26.68)	78 (27.37)	- (11.76)	
*70–79*	286 (19.29)	83 (15.37)	136 (21.22)	62 (21.75)	- (29.41)	
≥*80*	65 (4.38)	18 (3.33)	31 (4.84)	16 (5.61)	0	0.073
Sex (male)	1164 (78.49)	448 (82.96)	483 (75.35)	217 (76.14)	16 (94.12)	0.003
Rural residence^¶^	80 (5.39)	24 (4.44)	46 (7.18)	10 (3.51)	0	0.085
Income quintile^¶^						
*1 (lowest)*	391 (26.37)	171 (31.67)	153 (23.87)	64 (22.46)	- (17.65)	
*2*	343 (23.13)	106 (19.63)	156 (24.34)	79 (27.72)	- (11.76)	
*3*	289 (19.49)	110 (20.37)	127 (19.81)	47 (16.49)	- (29.41)	
*4*	250 (16.86)	80 (14.81)	114 (17.78)	50 (17.54)	6 (35.29)	
*5 (highest)*	204 (13.76)	71 (13.15)	88 (13.73)	44 (15.44)	- (5.88)	0.033
Charlson-Deyo Comorbidity Index						
*0*	686 (46.26)	266 (49.26)	284 (44.31)	130 (45.61)	6 (35.29)	
*1*	196 (13.22)	74 (13.70)	85 (13.26)	36 (12.63)	- (5.88)	
*2*	141 (9.51)	68 (12.59)	49 (7.64)	22 (7.72)	- (11.76)	
*3 or more*	64 (4.32)	25 (4.63)	29 (4.52)	10 (3.51)	0	
*No hospitalisation*	396 (26.70)	107 (19.81)	194 (30.27)	87 (30.53)	8 (47.06)	0.002
Diabetes diagnosis	624 (42.08)	218 (40.37)	280 (43.68)	121 (42.46)	- (29.41)	0.483
Indicators of severe liver disease						
*No ALD + No cirrhosis*	710 (47.88)	221 (40.93)	333 (51.95)	145 (50.88)	11 (64.71)	0.001
*No ALD + Cirrhosis only*	215 (14.50)	71 (13.15)	95 (14.82)	49 (17.19)	0	0.144
*No ALD + Decompensated cirrhosis* ^§^	350 (23.60)	143 (26.48)	134 (20.90)	70 (24.56)	- (17.65)	0.138
*ALD + No cirrhosis*	11 (0.74)	6 (1.11)	- (0.47)	- (0.70)	0	0.514
*ALD + Cirrhosis*	47 (3.17)	22 (4.07)	21 (3.28)	- (1.05)	- (5.88)	0.056
*ALD + Decompensated cirrhosis* ^§^	134 (9.04)	70 (12.96)	51 (7.96)	11 (3.86)	- (11.76)	<0.001
*NAFLD + Cirrhosis*	16 (1.08)	7 (1.30)	- (0.62)	5 (1.75)	0	0.359
Outpatient visits in 2 years before HCC diagnosis, mean (SD)	43.2 (31.15)	44.33 (35.13)	42.28 (30.00)	43.06 (25.65)	44.53 (22.86)	0.507
HCC treatment						
*Curative*	688 (46.39)	217 (40.19)	292 (45.55)	168 (58.95)	11 (64.71)	
*Non-curative*	156 (10.52)	49 (9.07)	77 (12.01)	27 (9.47)	- (17.65)	
*Palliative*	332 (22.39)	140 (25.93)	146 (22.78)	45 (15.79)	- (5.88)	
*No treatment*	307 (20.70)	134 (24.81)	126 (19.66)	45 (15.79)	- (11.76)	<0.001
Year of HCC diagnosis						
*2000–2001*	173 (11.67)	52 (9.63)	75 (11.70)	44 (15.44)	- (11.76)	
*2002–2003*	199 (13.42)	62 (11.48)	88 (13.73)	47 (16.49)	- (11.76)	
*2004–2005*	270 (18.21)	100 (18.52)	124 (19.34)	42 (14.74)	- (23.53)	
*2006–2007*	303 (20.43)	115 (21.30)	133 (20.75)	52 (18.25)	- (17.65)	
*2008–2009*	347 (23.40)	131 (24.26)	151 (23.56)	62 (21.75)	- (17.65)	
*2010*	191 (12.88)	80 (14.81)	70 (10.92)	38 (13.33)	- (17.65)	0.268

‘‘-‘‘, counts less than six have been suppressed.

*At least 1 screen either within 12 months or between 12-<24 months before HCC diagnosis

^†^1 screen annually for 2 years before HCC diagnosis

^‡^≥2 screens annually for 2 years before HCC diagnosis.

^¶^Missing data: rural residence (n = 2); Income quintile (n = 6).

^§^Decompensated cirrhosis: i.e., cirrhosis and any recorded ascites, esophageal varices, or hepatic encephalopathy.

**Fig 2:**
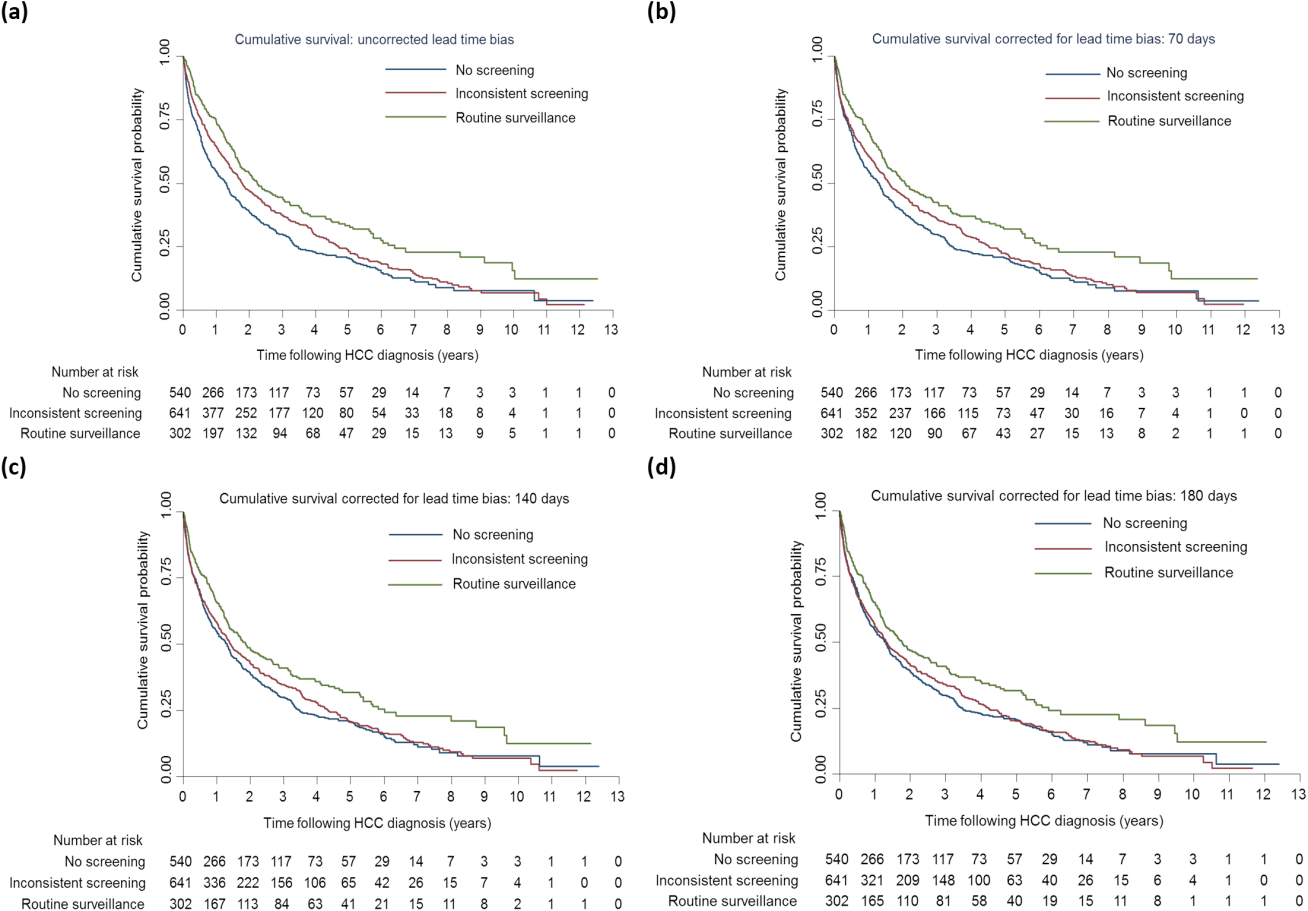
Kaplan-Meier survival estimates of patients diagnosed with viral hepatitis-induced hepatocellular carcinoma by the timing of ultrasonographic surveillance*, 2000–2010: 2a (uncorrected for lead-time bias); 2b (lead-time bias corrected with HCC sojourn time 70 days); 2c (lead-time bias corrected with HCC sojourn time 140 days); 2d 2b (lead-time bias corrected with HCC sojourn time 180 days). *Routine surveillance: 1 screen annually and ≥2 screens annually for 2 years before HCC diagnosis. Inconsistent screening: at least 1 screen either within 12 months or between 12-<24 months before HCC diagnosis.

The unadjusted median survival after HCC diagnosis among those who received routine surveillance, inconsistent screening, and no screening was 821 days (27.0 months), 652 days (21.4 months), and 478 days (15.7 months), respectively (Table 2). The respective 3-year survival rates were 44.5%, 37.5%, and 29.9%; and the respective 5-year survival rates were 33.4%, 23.3%, and 20.7%. The median survival after HCC diagnosis of routine surveillance and inconsistent screening corrected for lead-time bias (HCC sojourn times of 70/140 days) was: 751/679 days (24.7/22.3 months) and 582/515 days (19.1/16.9 months), respectively; the respective corrected 3-year survival rates were 42.6%/41.1% and 35.7%/34.8.0% and the respective corrected 5-year survival rates were 31.9%/31.8% and 22.4%/20.5%. There were significant differences in median survival (corrected for lead-time bias up to 140 days) between routine surveillance and no screening and in cumulative survival (corrected lead-time bias up to 180 days) between routine surveillance and no screening (log-rank test: *p*<0.001) as well as between routine surveillance and inconsistent screening (log-rank test: *p* = 0.002) (Fig 2A–2D).

**Table 2:** Observed (uncorrected) and lead time bias corrected median survival times and cumulative survival following hepatocellular carcinoma diagnosis among patients with viral hepatitis by different timing of ultrasonographic surveillance (N = 1,483).

Screening	N (%)	Median survival, days(95% CI)	1-year survival(%) (95% CI)	3-year survival(%) (95% CI)	5-year survival(%) (95% CI)
**Uncorrected for lead time bias**					
*Routine surveillance* *	302 (20.36)	821 (643, 1105)	74.56 (69.01, 79.27)	44.54 (38.33, 50.57)	33.42 (27.27, 39.66)
*Inconsistent screening*	641 (43.22)	652 (580, 770)	64.72 (60.78, 68.37)	37.52 (33.50, 41.54)	23.28 (19.53, 27.22)
*No screening*	540 (36.41)	478 (371, 523)	54.67 (50.25, 58.87)	29.88 (25.77, 34.09)	20.67 (16.86, 24.74)
**Corrected for lead time bias**					
Sojourn time = 42 days					
*Routine surveillance* *	302 (20.36)	779 (601, 1063)	71.46 (65.73, 76.41)	42.61 (36.41, 48.67)	32.68 (26.53, 38.95)
*Inconsistent screening*	641 (43.22)	610 (525, 728)	62.09 (58.09, 65.82)	36.63 (32.61, 40.64)	22.38 (18.67, 26.31)
Sojourn time = 70 days					
*Routine surveillance* *	302 (20.36)	751 (573, 1035)	70.26 (64.46, 75.29)	42.58 (36.38, 48.64)	31.93 (25.77, 38.24)
*Inconsistent screening*	641 (43.22)	582 (497, 697)	60.67 (56.65, 64.44)	35.74 (31.74, 39.75)	22.36 (18.65, 26.29)
Sojourn time = 121 days					
*Routine surveillance* *	302 (20.36)	701 (525, 978)	66.63 (60.67, 71.91)	41.08 (34.9, 47.16)	31.87 (25.72, 38.17)
*Inconsistent screening*	641 (43.22)	531 (451, 647)	58.85 (54.8, 62.67)	34.80 (30.82, 38.81)	20.49 (16.84, 24.39)
Sojourn time = 140 days					
*Routine surveillance* *	302 (20.36)	679 (500, 959)	65.81 (59.81, 71.13)	41.05 (34.86, 47.12)	31.84 (25.69, 38.14)
*Inconsistent screening*	641 (43.22)	515 (431, 630)	58.29 (54.23, 62.12)	34.77 (30.79, 38.77)	20.47 (16.83, 24.36)
Sojourn time = 180 days					
*Routine surveillance* *	302 (20.36)	646 (472, 921)	65.32 (59.29, 70.68)	40.97 (34.79, 47.04)	31.78 (25.64, 38.07)
*Inconsistent screening*	641 (43.22)	487 (407, 594)	56.11 (52.03, 59.99)	34.01 (30.04, 38.02)	20.10 (16.48, 23.99)
Sojourn time = 614 days					
*Routine surveillance* *	302 (20.36)	470 (363, 622)	55.84 (49.54, 61.68)	34.76 (28.64, 40.95)	22.76 (16.57, 29.56)
*Inconsistent screening*	641 (43.22)	371 (310, 432)	50.12 (45.97, 54.13)	26.92 (23.09, 30.88)	14.76 (11.42, 18.50)

CI, confidence interval.

*Includes 1 screen annually for 2 years before HCC diagnosis (n = 285) and ≥2 screens annually for 2 years before HCC diagnosis (n = 17).

In the unadjusted Cox proportional-hazards regression models with an assumed HCC sojourn time of 70 or 140 days, the association between routine HCC surveillance and mortality risk reduction was significant; inconsistent screening was, however non-significant if sojourn time was assumed to be at least 70 days (Table 3). In the fully adjusted Cox proportional-hazards regression model with correction for lead-time bias (HCC sojourn time of 70 days), those receiving routine surveillance or inconsistent screening before HCC diagnosis had a lower mortality risk (aHR 0.76, 95% CI: 0.64 to 0.91 and aHR 0.86, 95% CI: 0.75 to 0.98, respectively) than those who did not undergo screening. With the assumed HCC sojourn time of 140 days, receipt of routine surveillance was associated with a lower mortality risk (aHR 0.81, 95% CI: 0.68 to 0.97) compared with no screening; inconsistent screening was, however non-significant if sojourn time was assumed to be at least 121 days.

**Table 3:** Association between different timing of ultrasonographic surveillance and the risk of mortality following hepatocellular carcinoma diagnosis among patients with viral hepatitis: Cox proportional-hazards regression models, with survival times uncorrected and corrected for lead time bias.

Screening	UnadjustedHR (95% CI)	*P*-value	Age-sex adjusted HR (95% CI)	*P*-value	Fully adjusted*HR (95% CI)	*P*-value	Fully adjusted^†^HR (95% CI)	*P*-value
**Uncorrected for lead time bias**								
*Routine surveillance*	0.623 (0.523, 0.743)	<0.001	0.611 (0.512, 0.729)	<0.001	0.666 (0.555, 0.799)	<0.001	0.706 (0.589, 0.848)	<0.001
*Inconsistent screening*	0.819 (0.716, 0.936)	0.003	0.806 (0.704, 0.923)	0.002	0.833 (0.725, 0.956)	0.010	0.778 (0.677, 0.894)	<0.001
*No screening*	1.00 (referent)		1.00 (referent)		1.00 (referent)		1.00 (referent)	
**Corrected for lead time bias**								
Sojourn time = 42 days								
*Routine surveillance*	0.655 (0.549, 0.781)	<0.001	0.642 (0.538, 0.766)	<0.001	0.692 (0.577, 0.830)	<0.001	0.740 (0.617, 0.887)	0.001
*Inconsistent screening*	0.862 (0.754, 0.986)	0.030	0.849 (0.742, 0.972)	0.018	0.877 (0.764, 1.008)	0.064	0.830 (0.722, 0.953)	0.008
*No screening*	1.00 (referent)		1.00 (referent)		1.00 (referent)		1.00 (referent)	
Sojourn time = 70 days								
*Routine surveillance*	0.672 (0.563, 0.801)	<0.001	0.659 (0.552, 0.786)	<0.001	0.711 (0.592, 0.852)	<0.001	0.762 (0.635, 0.914)	0.003
*Inconsistent screening*	0.886 (0.775, 1.012)	0.075	0.872 (0.762, 0.998)	0.047	0.902 (0.785, 1.036)	0.142	0.856 (0.745, 0.983)	0.028
*No screening*	1.00 (referent)		1.00 (referent)		1.00 (referent)		1.00 (referent)	
Sojourn time = 121 days								
*Routine surveillance*	0.699 (0.586, 0.833)	<0.001	0.686 (0.574, 0.818)	<0.001	0.741 (0.618, 0.888)	0.001	0.798 (0.665, 0.957)	0.015
*Inconsistent screening*	0.921 (0.805, 1.052)	0.226	0.907 (0.792, 1.038)	0.155	0.937 (0.816, 1.076)	0.357	0.895 (0.779, 1.029)	0.119
*No screening*	1.00 (referent)		1.00 (referent)		1.00 (referent)		1.00 (referent)	
Sojourn time = 140 days								
*Routine surveillance*	0.709 (0.595, 0.846)	<0.001	0.696 (0.583, 0.830)	<0.001	0.753 (0.628, 0.903)	0.002	0.812 (0.677, 0.974)	0.025
*Inconsistent screening*	0.935 (0.818, 1.069)	0.323	0.921 (0.804, 1.054)	0.230	0.951 (0.828, 1.093)	0.481	0.912 (0.794, 1.047)	0.191
*No screening*	1.00 (referent)		1.00 (referent)		1.00 (referent)		1.00 (referent)	
Sojourn time = 180 days								
*Routine surveillance*	0.727 (0.610, 0.866)	<0.001	0.713 (0.597, 0.851)	<0.001	0.773 (0.644, 0.927)	0.005	0.836 (0.697, 1.002)	0.053
*Inconsistent screening*	0.958 (0.838, 1.095)	0.528	0.943 (0.824, 1.08)	0.398	0.977 (0.850, 1.122)	0.740	0.938 (0.817, 1.078)	0.366
*No screening*	1.00 (referent)		1.00 (referent)		1.00 (referent)		1.00 (referent)	
Sojourn time = 614 days								
*Routine surveillance*	0.861 (0.722, 1.026)	0.095	0.846 (0.709, 1.009)	0.063	0.921 (0.768, 1.104)	0.373	1.002 (0.836, 1.202)	0.980
*Inconsistent screening*	1.134 (0.992, 1.297)	0.065	1.118 (0.977, 1.280)	0.105	1.161 (1.010, 1.334)	0.036	1.125 (0.979, 1.292)	0.098
*No screening*	1.00 (referent)		1.00 (referent)		1.00 (referent)		1.00 (referent)	

*Adjusted for: age at HCC diagnosis; sex; rural residence; income quintile; Charlson-Deyo Comorbidity Index; diabetes diagnosis; indicators of severe liver disease: No alcoholic liver disease (ALD)+no cirrhosis; No ALD+Cirrhosis only; No ALD+Decompensated cirrhosis; ALD+No cirrhosis; ALD+Cirrhosis; ALD+Decompensated cirrhosis; Non-alcoholic fatty liver disease (NAFLD)+Cirrhosis; and index year of hepatocellular carcinoma (HCC) diagnosis.

^†^All covariates, including receipt of HCC curative treatment (i.e., surgical resection, liver transplantation, or radiofrequency ablation). Variables modeled as time-dependent covariate include: Charlson-Deyo Comorbidity Index; diabetes diagnosis; and HCC curative treatment.

In the univariate analysis determining predictors of receiving ≥1 ultrasound screening annually for 2 years before HCC diagnosis (routine surveillance) among patients with viral hepatitis, rural residence (p = 0.091), no ALD +no cirrhosis (p = 0.141), ALD+cirrhosis (p = 0.064), ALD+decompensated cirrhosis (p = 0.003), and outpatient visits (p = 0.001) were significant variables (Table 4). In the multivariable log binomial regression analysis, ALD+cirrhosis (aRR 0.38, 95% CI: 0.15 to 0.96, p = 0.042) and ALD+decompensated cirrhosis (aRR 0.44, 95% CI: 0.26 to 0.74, p = 0.002) were associated with decreased odds of receiving routine surveillance, whereas outpatient visits (21–40 vs. 0–20 visits: aRR 1.74, 95% CI: 1.26 to 2.39, p = 0.001 and >40 vs. 0–20 visits: aRR 1.65, 95% CI: 1.20 to 2.27, p = 0.002) were associated with increased odds of receiving routine surveillance.

**Table 4:** Predictors of receiving one or more ultrasound screening annually for 2 years before hepatocellular carcinoma diagnosis among patients with viral hepatitis: Log binomial regression.

Characteristic	Unadjusted RR (95% CI)	*P*-value	Adjusted RR (95% CI)	*P*-value
Age at HCC diagnosis (years)*				
*<60*	1.00 (referent)			
*60–69*	0.998 (0.780, 1.278)	0.988	-	-
*70–79*	1.210 (0.936, 1.565)	0.146	-	-
≥*80*	1.271 (0.810, 1.996)	0.296	-	-
Sex (male vs. female)	0.925 (0.729, 1.175)	0.524	-	-
Rural residence (yes vs. no)	0.602 (0.334, 1.084)	0.091	-	-
Income quintile^†^				
*1 (lowest)*	1.00 (referent)			
*2*	1.378 (1.032, 1.841)	0.030	-	-
*3*	1.050 (0.756, 1.459)	0.771	-	-
*4*	1.307 (0.952, 1.796)	0.098	-	-
*5 (highest)*	1.287 (0.918, 1.804)	0.143	-	-
Charlson-Deyo Comorbidity Index^‡^				
*0*	1.00 (referent)			
*1*	0.952 (0.687, 1.320)	0.769	-	-
*2*	0.859 (0.579, 1.274)	0.448	-	-
*3 or more*	0.788 (0.437, 1.420)	0.428	-	-
*No hospitalisation*	1.210 (0.960, 1.525)	0.106	-	-
Diabetes diagnosis (yes vs. no)	0.986 (0.804, 1.209)	0.889	-	-
Indicators of severe liver disease				
*No ALD + No cirrhosis*	1.163 (0.951, 1.423)	0.141	-	-
*No ALD + Cirrhosis only*	1.142 (0.872, 1.496)	0.334	-	-
*No ALD + Decompensated cirrhosis*	1.032 (0.816, 1.305)	0.793	-	-
*ALD + No cirrhosis*	0.892 (0.254, 3.138)	0.859	-	-
*ALD + Cirrhosis*	0.410 (0.160, 1.053)	0.064	0.375 (0.146, 0.963)	0.042
*ALD + Decompensated cirrhosis*	0.453 (0.267, 0.767)	0.003	0.436 (0.257, 0.738)	0.002
*NAFLD + Cirrhosis*	1.544 (0.741, 3.215)	0.246	-	-
Outpatient visits in 2 years before HCC diagnosis^¶^				
*0–20 visits*	1.00 (referent)			
*21–40 visits*	1.735 (1.260, 2.389)	0.001	1.739 (1.264, 2.391)	0.001
*>40 visits*	1.574 (1.143, 2.166)	0.005	1.653 (1.202, 2.273)	0.002
Viral hepatitis index year^¥^				
*2000–2001*	1.00 (referent)			
*2002–2003*	1.107 (0.704, 1.738)	0.660	-	-
*2004–2005*	1.328 (0.798, 2.209)	0.275	-	-
*2006–2007*	1.324 (0.699, 2.505)	0.389	-	-

Overall *p*-values

*Age at HCC diagnosis: *p* = 0.365 (unadjusted)

^†^Income quintile: *p* = 0.243 (unadjusted)

^‡^Charlson-Deyo Comorbidity Index: *p* = 0.2436 (unadjusted)

^¶^Outpatient visits in 2 years before HCC diagnosis: *p* = 0.001 (unadjusted); *p* = 0.002 (adjusted)

^¥^Viral hepatitis index year: *p* = 0.673 (unadjusted).

RR, risk ratio; CI, confidence interval; HCC, hepatocellular carcinoma; ALD, alcoholic liver disease; NAFLD, non-alcoholic fatty liver disease.

## Discussion

In this population-based retrospective cohort study examining the timing of ultrasonographic surveillance in patients diagnosed with viral hepatitis-induced HCC in Ontario and their impact on survival and mortality, approximately 20% of patients received routine surveillance (i.e., ≥2 screens or 1 screen annually for 2 years before HCC diagnosis). The proportion of patients receiving potentially curative treatment was higher among those under routine surveillance compared to those receiving inconsistent screening or no screening at all. The results indicate significant improvements in cumulative survival after an HCC diagnosis among patients receiving routine surveillance when corrected for lead-time bias. Furthermore, those receiving routine surveillance had a lower mortality risk, adjusted for important factors, with an average of approximately 21% (range: 5–34%). In the multivariable log binomial regression analysis, high-risk viral hepatitis patients with ALD and cirrhosis or decompensated cirrhosis are associated with decreased odds of receiving routine surveillance before HCC diagnosis.

Our findings suggest that ultrasonographic surveillance may increase survival among patients with viral hepatitis-induced HCC in real world clinical practice. As Canada’s most populated province, Ontario is generally representative of the country with a single-payer healthcare system and universal access to health services. Our findings are consistent with other published studies including a randomized controlled trial of 18,816 HBV-infected individuals in urban Shanghai, China, where surveillance with alpha-fetoprotein (AFP) and abdominal ultrasounds were performed every 6 months and were associated with a 37% (range 2–59%) reduction in HCC-related mortality [23]. Furthermore, a cohort study by El-Serag *et al*. [54] examining the effectiveness of HCC surveillance with AFP and abdominal ultrasound on mortality in HCV-infected patients found that routine surveillance (6-monthly) was independently associated with a reduced mortality risk (20–30%) when corrected for lead-time with an assumed HCC sojourn time of 70 days. However, when sojourn time was assumed to be 140 days, the association between routine surveillance and mortality became non-significant. In our study, routine surveillance among patients with viral hepatitis was independently associated with a reduced mortality risk for an assumed sojourn time of 42 days to 180 days. When the application of time-dependent HCC curative treatment was made, the protective effect of surveillance was moderately attenuated, which is similar to the study by El-Serag *et al*. [54] This may be due to poor liver function that adversely affects overall survival and ability to undergo HCC treatment [55] but it may also suggest that if HCC sojourn time is long, then routine surveillance duration needs to be shorter than 6 months in order to positively affect survival from a cancer disease progression perspective. In terms of possible factors that may determine which patients are more likely to receive recommended ultrasound surveillance, not surprisingly, patients who had fewer out-patient clinic visits were less likely to undergo recommended surveillance, which most likely reflects suboptimal patient adherence to medical recommendations. The fact that alcoholic liver disease (in the setting of viral hepatitis) was also associated with a lesser likelihood of receiving recommended surveillance may also be linked to suboptimal healthcare or poor adherence to healthcare follow-up although some may also be healthcare provider bias. This may be an area where the healthcare profession can improve patient outcomes.

Viral-hepatitis associated disease and progression to HCC accounts for a significant medical burden in both Canada and around the world. HCC is amenable to a number of potentially curative treatments in the context of early detection. Despite this, our study identifies that a minority of viral hepatitis-induced HCC patients received routine ultrasonographic surveillance. Indeed, rigorous surveillance has a significant impact on survival post-HCC diagnosis, likely due to increased use of the aforementioned treatment options. Routine implementation of surveillance strategies may have benefits outside of patient wellbeing by avoiding the significant financial burden of HCC disease management in advanced cases, a pertinent issue in publically-funded universal healthcare systems such as Canada's. The implementation of these suggested surveillance practices may prove to be cost-effective for a disease that requires such significant public resources.

Our study has several limitations and its results should be interpreted cautiously. First, due to lack of information on AFP screening, our study assessed for abdominal ultrasound screening only. A recent study evaluating the effectiveness of HCC surveillance programs in patients with cirrhosis in a real-world clinical setting found that a combination of ultrasonography and AFP is the most effective strategy to detect early-stage HCC [56]. Nevertheless, a recent meta-analysis of prospective cohort studies [57] and a nested case-control study [58] showed that AFP determination lacks adequate sensitivity and specificity for effective surveillance and diagnosis [57,58]. Consequently, AASLD and multidisciplinary Canadian consensus practice guidelines recommend an ultrasound-based surveillance strategy which is more sensitive than serology markers even in patients with cirrhosis [30,59]. Second, although health administrative data can be used to examine the timing of ultrasound tests, they are unable to distinguish the reason the test was performed, i.e., between imaging performed for HCC surveillance in asymptomatic patients and those performed in symptomatic patients for HCC diagnosis (i.e., clinical suspicion) or for non-HCC issues [31,33,54,60]. To address this issue, we applied criteria to reduce the number of ultrasounds counted as HCC surveillance that were actually diagnostic tests or were performed for non-HCC related purposes. For example, some criteria for identifying potential HCC diagnostic tests could be a second ultrasound performed less than 6-months after the first ultrasound or an ultrasound performed at the same time as another imaging study (see S1 Table). However, it may be possible that the criteria for this study included imaging studies that were unrelated to surveillance. Third, our databases lacked information pertaining to cancer staging, HCC size, liver fibrosis staging etc., and thus we were unable to determine an optimal surveillance strategy related to the relative progression of HCC disease. Fourth, we were unable to capture nonpatient-specific factors such as facility characteristics (including geographic region or the availability of HCC treatment centers), physician recommendations to perform HCC surveillance, or patient adherence to surveillance. It is possible that there is a confounding reason that patients with a better prognosis may have been more likely to undergo screening. Fifth, HCC surveillance is recommended only in patients with HCV-induced cirrhosis (and in some instances in patients with advanced hepatic fibrosis) and in patients with non-cirrhotic HBV infection [10]. Therefore, the results of this study cannot be generalized to populations with a different composition in terms of severity of liver disease, including patients with less advanced disease. This study may gauge the application of curative treatments such as surgery, thus "falsely" increasing the value of surveillance. Finally, the timing of diagnosis of viral hepatitis or cirrhosis based on administrative claims may not be precise, however, our suspicion is that our conservative methodology would have underestimated any benefit rather than exaggerated it.

We note that given the consistent published recommendations of the hepatology professional organizations (i.e., AASLD, EASL) advocating screening, it is highly unlikely that a prospective, long-term randomized clinical trial would ever be funded and performed. Indeed, such a trial, assuming that the “control” arm would be offered less than recommended screening, most likely would be perceived to be unethical by both the hepatology community and institutional ethics review boards. Therefore, studies such as ours assume greater importance in answering the question of the value of screening.

### Conclusions

Our findings suggest that routine ultrasonography of the liver in patients with viral hepatitis before HCC diagnosis may be associated with a significant reduction in overall mortality. We suspect that HCC survival is improving because high-risk patients (including those with viral hepatitis) who have been diagnosed in recent years are receiving routine ultrasonographic surveillance, and those receiving surveillance are likely to receive potentially curative treatment. Further improvement may be possible with increased community-based surveillance to follow individuals at risk for HCC, enabling early intervention and mitigation of the burden of disease. On-going study of this subject will continue to be necessary.

## Supporting Information

S1 Table Exclusion criteria of fee codes (considering diagnostic tests or non-HCC related purposes) billed on the same days of abdominal ultrasound screening for HCC. (DOCX)
