# Supplementary material for: Improved Survival in Patients with Viral Hepatitis-Induced Hepatocellular Carcinoma Undergoing Recommended Abdominal Ultrasound Surveillance in Ontario: A Population-Based Retrospective Cohort Study
Source: PLoS One. 2015 Sep 23;10(9):e0138907. doi: 10.1371/journal.pone.0138907 (PMC4580446; doi:10.1371/journal.pone.0138907)
Supplement: S1 Table — (DOCX) [file pone.0138907.s001.docx]

S1 Table. Exclusion criteria of feecodes (considering diagnostic tests or non-HCC related purposes) billed on the same days of abdominal ultrasound screening for HCC

| **Feecode** | **Description** |
| --- | --- |
| A015 | CONSULT.-ANAES. |
| A034 | PARTIAL-ASSESS. -GEN. SURG. |
| A035 | CONSULT.-GEN. SURG. |
| A036 | RE-CONSULT.-GEN. SURG. |
| A084 | PARTIAL-ASSESS. -PLASTIC SURG. |
| A094 | PARTIAL-ASSESS. -CARDIO. & THOR. SURG. |
| A203 | SPECIFIC ASSESS.-OBS.& GYN. |
| A245 | CONSULT.-OTO. |
| A335 | CONSULT.-DIAG. RADIOLOGY |
| A353 | SPECIFIC ASSESS.-UROL. |
| A354 | PARTIAL ASSESS. -UROL. |
| A355 | CONSULT.-UROL. |
| A418 | PARTIAL ASSESS. -GASTROENT. |
| A603 | MEDICAL SPECIFIC ASSESSMENT |
| A608 | PARTIAL ASSESS. -CARDIOLO. |
| A888 | PARTIAL ASSESSMENT EM.DEPT EQUIVALENT |
| A901 | GENERAL/FAMILY PRACTICE-HOUSECALL ASSESSMENT |
| A903 | GEN/FAM PRACT-PRE-DENTAL/OPER.ASSESS LIMIT 2 PER YEAR/PT |
| A935 | PREAMBLE-SPECIAL SURGICAL CONSULT. |
| C002 | SUBSEQ.VISITS-TO 5WKS-F.P./G.P.-HOSP. |
| C004 | GENERAL RE-ASSESS-F.P./G.P.-HOSP. |
| C010 | SUPPORT CARE-F.P./G.P.-HOSP. |
| C032 | SUBSEQ VISIT UP TO FIVE WEEKS.- GEN. SURG. - HOSPITAL |
| C035 | CONSULT-GEN SURG-HOSPITAL |
| C101 | VISITS TO I.C.U./C.C.U. (EXTRA) |
| C109 | SPEC VIS NON-ELECT DIAG. & THERAP. PROC., MON-FRI, EVE |
| C122 | MOST RESPONSIBLE PHYSICIAN |
| C123 | MOST RESPONSIBLE PHYSICIAN |
| C124 | DAY OF DISCHARGE VISIT - MOST RESPONSIBLE PHYS |
| C132 | SUBSEQ. VISITS -UP TO 5 WKS. - INTERNAL MED. - HOSPITAL |
| C133 | MEDICAL SPECIFIC ASSESSMENT |
| C135 | CONSULT.-INTERNAL MED.-HOSPITAL |
| C138 | CONCURRENT CARE - INTERNAL MED. - HOSPITAL |
| C335 | DIAG.RADIOLOG.CONSULTATION.NON.EMERG.HOSP. |
| C412 | SUBSEQ.VISITS UP TO FIVE WKS.-GASTRO ENTEROLOGY-HOSPITAL |
| C415 | CONSULT.-GASTRO ENTEROLOGY-HOSPITAL |
| C416 | REPEAT CONSULT.-GASTRO ENTEROLOGY-HOSPITAL |
| C418 | CONCURRENT CARE-GASTRO ENTEROLOGY-HOSPITAL |
| C475 | CONSULT.-RESPIRATORY DISEASE-HOSPITAL |
| C478 | CONCUR.CARE-RESPIRATORY DISEASE-HOSPITAL |
| C903 | GENERAL ASSESS.PRIOR TO DENTAL SURGERY-HOSP. |
| C933 | ON-CALL ADMISSION GENERAL ASSESSMENT |
| C990 | SPEC VIS - HOSP IN-PT.- WK/DAYTIME |
| C995 | SPEC VIS - HOSP IN-PT., MON-FRI., EVE., ADDIT'L PT |
| E017 | PATIENTS ASA 4 - PATIENT WITH INCAPACITATING |
| E020 | ANAES ASA EMERG PATIENT PREMIUM (APPLIC ASA III, IV & V PTS |
| E022 | PATIENTS ASA 3 |
| E083 | SUBSEQ VIS MRP TO SUBSEQ VIS, C122/C123/C124/C142 OR C143 |
| E400 | ANAES/ASSISS.(5PM-12MN)EVE./SAT/SUN/HOL.EXTRA 40% |
| E409 | PROC.COMM.AFT.5PM BEF.12MN.EV/SAT/SUN/HOLS-PHYS.ADD 30% |
| E475 | PORTABLE U.S. BEDSIDE / O.R.PER 1/4 HR. ADD.TO U.S. FEE |
| E702 | OESOPH/GASTRO/DUODENOSCOPY MULT.BIOPSY 3 OR MORE ADD |
| E740 | INTESTINE ENDO SIGMOID TO SPLENIC FLEXURE ADD |
| E741 | INTESTINE END SIGMOID TO HEPATIC FLEXURE ADD |
| E747 | INTESTINE-ENDOSCOPY-SIGMOID.TO CAECUM ADD TO Z512/Z555 |
| E797 | ENDOSCOPY-UNCOMPLICATED UPP OR LOW GI BLEEDING-ADD |
| H055 | EMERGENCY DEPARTMENT - PHYSICIAN ON DUTY |
| H065 | EMERG.PHYS.CONSULT.(NON SPEC. .IN EMERG.MED.) |
| H101 | MINOR ASSESS.-G.P.-EMERG.-DR.ON DUTY. |
| H102 | COMPREHENSIVE ASSESS. & CARE |
| H103 | MULTIPLE SYSTEMS ASSESSMENT-G.P.-EMERGENCY-DR. ON DUTY |
| H104 | GP-REASSESS-EMERG DEPT-PHYSICIAN ON DUTY M-F DAYS |
| H105 | INTERIM INPATIENT ADMISSION ORDERS |
| H122 | 12 MIDNIGHT TO 8:00 A.M. COMPREHENSIVE ASSESS. & CARE |
| H123 | EMERG.DEPT.PHYS.ON DUTY 12MN-8AM MULT.SYST.ASSESS. |
| H124 | EMERG.DEPT.PHYS.ON DUTY 12MN-8AM RE-ASSESS. |
| H132 | PHYSICIAN ON DUTY IN ER - EVENINGS (18:00-24:00) |
| H133 | PHYSICIAN ON DUTY IN ER - EVENINGS (18:00-24:00) |
| H134 | GP-REASSESS-EMERG DEPT-PHYSICIAN ON DUTY M-F EVENINGS |
| H151 | EMERG.DEPT.PHYS.ON DUTY SAT./SUN./HOLIDAY MINOR ASSESS. |
| H152 | SAT/SUN & HOLIDAYS COMPREHENSIVE ASSESS. & CARE |
| H153 | EMERG.DEPT.PHYS.ON DUTY SAT./SUN/HOLIDAY MULT.SYST.ASSESS. |
| H154 | EMERG.DEPT.PHYS.ON DUTY SAT./SUN./HOLIDAY REASSESS. |
| J138 | D&T,ULTRASOUND-INTRACAVITY ULTRA SOUND(TRANSRECTAL/VAG) |
| J149 | DIAG.ULTRASOUND GUIDE BIOPSY/ASP/AMNIO/DRAINAGE |
| J162 | D&T,ULTRASOUND-PELVIC COMP. |
| J163 | PELVIS, LIMITED STUDY OTHER THAN PREGNANCY |
| J193 | DIAG.ULTRA SOUND PERIPH.ARTERY AND/OR VEIN EVALU'N |
| J201 | DIAG.ULTRASOUND-DUPLEX SCAN SIMULT.REAL TIME.B MODE VASC.SYS |
| J202 | DIAG.ULTRASOUND-DUPLEX SCAN SIMULT.REAL TIME.B MOD.PERIP.ART |
| J205 | DIAG.ULTRA SOUND-DOPPLER EVALU'N ORG.TRANSP'T-ARTER.&/ VEN. |
| J206 | DIAG.ULTRA SOUND-P1-DUPLEX EVALU'N (REQUESTED BY REF.DR) |
| J438 | D&T,ULTRA SOUND-PELVIS-P2 INTRACAVITY U.S.(TRANSRECT/VAG)1PE |
| J462 | DIAG.ULTRA SOUND-PELVIS-P2,COMPLETE 1 PER CYCLE * |
| J463 | PELVIS, LIMITED STUDY OTHER THAN PREGNANCY |
| J502 | DIAG.ULTRASOUND-VAS.SYST-P2-DUP.SCAN B-MODE/ANALYSIS...UNILA |
| K014 | COUNSELLING-FOR DONOR/RECIP/RECIP FAMILIES RE;ORGAN TRANSPLA |
| K990 | SPEC VIS EMERG DEPT., WK/DAYTIME |
| K991 | SPEC VIS EMERG DEPT., WK/DAYTIME, ADDIT'L PT. |
| K992 | SPEC VIS EMERG DEPT.-SAC.OFF.HRS. WK/DAYTIME |
| K993 | SPEC VIS EMERG DEPT.-SAC.OFF.HRS. WK/DAYTIME ADDIT'L PT. |
| K994 | SPEC VIS - EMERG DEPT., MON-FRI., EVE. |
| K995 | SPEC VIS - EMERG DEPT., MON-FRI., EVE. ADDIT'L PT. |
| K996 | SPEC VIS - EMERG DEPT., NIGHTS |
| K997 | SPEC VIS - EMERG DEPT., NIGHTS ADDIT'L PT. |
| P004 | OBS.-PRENATAL CARE-MINOR PRENATAL ASSESS.-SUBSEQ.PRENAT.VIS. |
| Q012 | AFTER HOURS PREMIUM |
| Q016 | AFTER HOURS PREMIUM - CCM |
| S287 | BILIARY TRACT EXC.-CHOLECYSTECTOMY |
| X100 | DIAG.RADIOLOGY ABDOMEN SINGLE VIEW |
| X101 | DIAG.RADIOLOGY ABDOMEN TWO/MORE VIEWS |
| X104 | DIAG. RADIOL.OESOP.STOM. & DUO.-SURVEY FILM-DOUBLE CONTRAST |
| X108 | DIAGNOSTIC RADIOLOGY-OESOPHAGUS, STOMACH, & DUODENUM |
| X113 | DIAG.RADIOLOGY COLON-AIR CONTRAST PRIMAR/SECOND/INCL.SURVEY |
| X126 | DIAGNOSTIC RADIOLOGY-ABDOMEN-WITH /OUT I.V. CONTRAST C.T.T. |
| X231 | DIAG.RAD.-X-RAY-PELVIS WITH I.V.CONTRAST |
| X232 | DIAG.RAD.-PELVIS WITH I.V. CONTRAST |
| X233 | DIAG RAD.-X-RAY PELVIS WITH/OUT I.V. CONTRAST |
| X400 | DIAG.RADIOLOGY-COMPUTED TOMOGRAHHY-HEAD-WITHOUT I.V.CONTRAST |
| X409 | DIAG. RADIOLOGY-COMPUTED TOMOGRAPHY-ABDOMEN-W'OUT I.V.CONTR. |
| X410 | DIAG. RADIOLOGY-COMPUTED TOMOGRAPHY-ABDOMEN-WITH I.V.CONTR. |
| Z153 | SKIN.DEBRIDE&DRESS'G MAJOR(NOT WITH Z176) |
| Z399 | OESOPHAGUS-OESOPHAGO/GASTRO. WITH/OUT DUODENOSCOPY |
| Z400 | OESOPHAGOSCOPY-GASTROCOPY- &/NO DUODENOSCOPY ACTIVE BLEEDING |
| Z459 | NUCLEAR MED-IN VIVO-ARTERIAL PUNCTURE. |
| Z551 | LIVER-INCISION-BIOPSY,NEEDLE |
| Z555 | INTESTINES-ENDOSCOPY-COLONOSCOPY INTO DESCENDING COLON |
| Z560 | INTESTINES-DUODENOSCOPY |
| Z571 | INTESTINES-EXC.-POLYPS THRO. COLONOSCOPE |
| Z590 | ABD.PERITONEUM.OMENTUM-PARACENTESIS-ASPIRATION DIAG. SAMPLE |
| Z591 | ABD.PERITONEUM,OMENTUM-PARACENTESIS-THERAPEUT.DRAIN. SAMPLE |
| Z601 | KIDNEY,PERINEPHRIUM-INC.-RENAL BIOPSY, NEEDLE |
| Z606 | BLADDER-ENDO/CYSTOSCOPY-DIAG.PROC.-DIAG.WITH/WITHOUT URETHRO |
